# Possible Advantages of Minimal-Invasive Approaches in Rectal Cancer Surgery: A Nationwide Analysis

**DOI:** 10.3390/jcm12144765

**Published:** 2023-07-19

**Authors:** Philipp Horvath, Christoph Steidle, Can Yurttas, Isabella Baur, Alfred Königsrainer, Ingmar Königsrainer

**Affiliations:** 1 Department of General, Visceral and Transplant Surgery, Comprehensive Cancer Center, University of Tübingen, 72074 Tübingen, Germany; 2 Department of General, Visceral and Thoracic Surgery, Academic Teaching Hospital Feldkirch, Carinagasse 47, 6807 Feldkirch, Austria

**Keywords:** rectal cancer, minimal-invasive surgery, robotic, hospital mortality

## Abstract

(1) Background: Laparoscopic resection for colon and rectal cancer was introduced in the early 1990s; the aim of this analysis was to show possible advantages of minimal-invasive approaches in rectal cancer surgery. (2) Methods: From 2016 to 2020, all patients undergoing open, laparoscopic or robotic-assisted rectal cancer surgery in Germany were retrospectively analyzed regarding sex distribution, conversion rates and in-hospital mortality rates according to nationwide hospital billing data based on diagnosis-related groups (DRGs). (3) Results: In total, 68,112 patients were analyzed, and most commonly, low anterior rectal resections with primary anastomosis (*n* = 25,824) were performed with an increase of minimal-invasive procedures over the years (open: 51% to 27%; laparoscopic: 47% to 63% and robotic: 2% to 10%). In-hospital mortality rate was 2.95% (*n* = 2012). In total, 4.61%, 1.77%, 1.14% and 3.95% of patients with open, laparoscopic, robotic and converted-to-open surgery died during hospital stay, respectively (open vs. laparoscopic *p* < 0.0001; open vs. robotic *p* < 0.00001; laparoscopic vs. robotic *p* = 0.001). Conversion rates were significantly more favorable in the robotic compared to the laparoscopic group. (11.94% vs. 2.53%; *p* < 0.0001). (4) Conclusion: Minimal-invasive rectal cancer surgery might have some advantages in terms of a reduced in-hospital mortality, and an improved conversion rate for the robotic approach.

## 1. Introduction

In the early 1990s, the first publications describing the feasibility of laparoscopic colon resections were published [1,2,3]. Early data indicated enhanced benefits of the minimal-invasive approach regarding postoperative pain, recovery, time to first flatus and oral intake [4,5,6,7,8]. Subsequently, the surgical society wanted to address the question whether or not the laparoscopic approach is noninferior to open colorectal resections for carcinoma. Four large randomized controlled trials did not show significant differences in overall and disease-free survival after 3- and 5-years follow-up [9,10,11,12,13,14,15,16,17]. When interpreting these data, two remarks have to be considered. The first one is that in all four clinical trials, there were a variety of in- and exclusion criteria and the operating surgeons had to have a completed learning curve for minimal-invasive colorectal procedures, which surely does not reflect the true medical care situation in almost all countries. The second fact is that the vast majority of patients in the named randomized trials were recruited before the concept of complete mesocolic excision (CME) and total mesorectal excision (TME) were universally applied. Nevertheless, these data depicted the noninferiority of the laparoscopic approach in terms of overall and disease-free survival; the transfer of these results into daily practice proceeded very slowly. A German nationwide analysis, published in 2018, summarizing all colorectal resections from 2005 to 2015, showed that the rate of minimal-invasive colorectal surgeries increased from 6.4% in 2005 to 28.5% in 2015. The vast majority of patients scheduled for a laparoscopic resection had left-sided colon or rectal cancer (38% and 39% of 345,913 patients, respectively) [18]. Interestingly, in this aforementioned publication, the mortality rate was significantly lower to the disfavor of the open approach (1.8% vs. 4.7%; *p* < 0.001). This is in line with other registry data showing a lower postoperative mortality rate in patients undergoing laparoscopic resection [19,20]. This slow adoption of laparoscopy may be explained by two closely linked issues. The first one is surely the intrinsic limitations of the equipment used during laparoscopy. Addison et al. summarized the major drawbacks of laparoscopic tools: nonangulated rigid instruments with only 4° of freedom, decreased depth perception and reduction of a three-dimensional anatomy to a two-dimensional plane [21]. Furthermore, visualization of critical structures, pivotal for preventing conversion to open surgery, is also dependent on the camera work of the assistant and the physique of the patient. In the AlCCas trial, the conversion rate was 14.6%, and in almost 5% the reason for conversion was inability to visualize critical structures, emphasizing the necessity of a very good assistant surgeon and the advantages of camera work during robotic-assisted surgeries [15]. All of these limitations may lead to a prolonged learning curve (approximately 62 cases for left-sided resection) associated with longer operating times at the beginning, and may be a cause for avoidance of this approach [21]. 

The development and introduction of robotic surgery devices equipped with angulated articulated instruments simulating natural wrist movements, combined with an augmented and magnified visualization, especially in the deep male pelvis, attempted to overcome these limitations of laparoscopic instruments. The most serious disadvantage of the robotic approach is still its cost-effectiveness, because the up-front costs range from USD 1.0 million to USD 2.5 million. Furthermore, average maintenance costs per year are USD 200,000. Simianu VV et al. stated in their manuscript that a robotic colectomy becomes cost-effective if robotic disposable instrument costs decrease below USD 1341 per case, robotic operating room time falls below 172 min or robotic hernia rate is less than 5% [22]. Meeting these demands is going to be a challenge for robotic-assisted surgeries in the future. Furthermore, the availability and effectiveness of robotic-assisted surgeries in the emergency setting are still under investigation. The WSES position paper, published in 2022, attempted to outline the prerequisites and contraindication for the use of robotic systems for urgent operations [23]. So far, the major limitations of this approach are surely the availability of trained surgeons and nursing staff, and the applicability only in clinically stable patients. 

In order to obtain insight into the current medical care situation of patients undergoing elective rectal surgeries for carcinoma in Germany, we evaluated the open, the laparoscopic, the robotic approach and the converted-to-open approach regarding caseload/year and in-hospital mortality and conversion rate.

## 2. Materials and Methods

From 2016 to 2020, all patients with the diagnosis *rectal cancer* (ICD 10 WHO version 2022: C20.) and the respective OPS-codes, indicating an open, laparoscopic or robotic approach, were evaluated. Data were harvested from the Federal Statistics Office. The OPS-codes 5-484.31/.32/.51/.52/.61 indicated open rectal cancer surgeries with or without primary anastomosis, but without involvement of the sphincter. The OPS-codes 5-484.35/.36/.55/.56/.65 indicated laparoscopic rectal cancer surgeries with or without primary anastomosis, but without involvement of the sphincter. OPS-codes indicating conversion to open surgery were the following: 5-484.38/.39/.58/.59/.68. The addition of the OPS-code 5-987.0 indicated the robotic approach. Rectal cancer surgeries performed as an abdominoperineal excision (APE) or extralevator abdominoperineal excision (ELAPE) were indicated by the following OPS-codes: 5-485.01 (open) and 5-485.02 (laparoscopic). The data were harvested from the Federal Statistic Office, and the provided data included caseload per year for each approach, sex distribution and the linked in-hospital mortality rates. Furthermore, conversion rates for the laparoscopic and the robotic approach were calculated. 

### Statistical Analysis 

Statistical analysis was performed using IBM SPSS software (version 25.0, IBM SPSS Inc., Chicago, IL, USA). All *p*-values were two-tailed, and a probability value of *p* < 0.05 was considered statistically significant. 

## 3. Results

**Figure 1:**
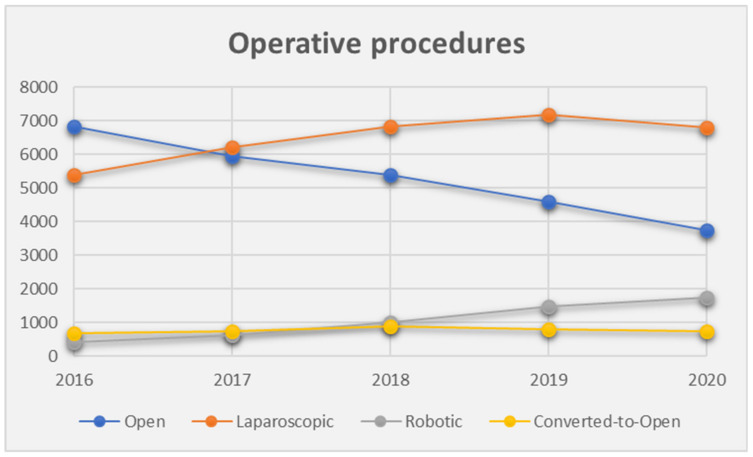
Dynamics of operative approaches over the study period.

**Figure 2:**
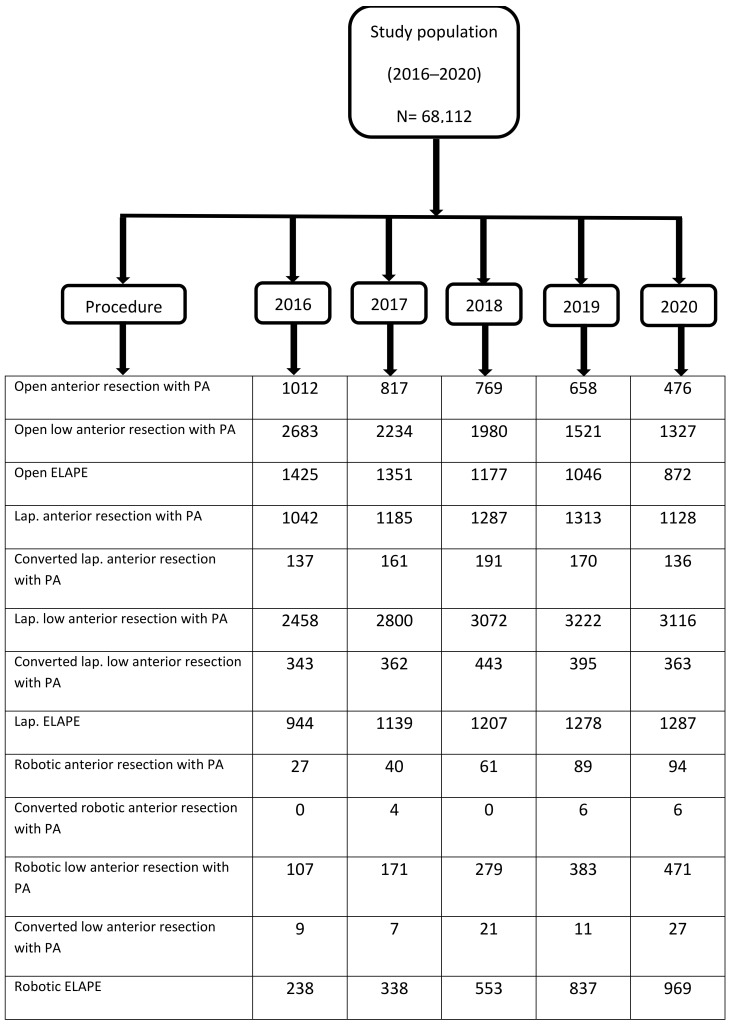
Performed procedures per year (sphincter-preserving procedures without primary anastomosis are not listed) (Lap. = laparoscopic; PA = primary anastomosis).

From 2016 to 2020, a total of 68,112 patients received open or minimal-invasive (laparoscopic or robotic) rectal cancer surgery. In total, 53,452 (78%) patients received sphincter-sparing resections, and 14,660 (22%) did not. Of the whole cohort, 63% (*n* = 43,224) were male. In 2018, the year with the highest caseload, 14,101 (21%) patients underwent surgery. In the first year of the COVID-19 pandemic, caseload dropped to 13,039 patients. Further characteristics are listed in Table 1, and Figure 1 and Figure 2.

The most commonly performed rectal cancer surgeries in all analyzed years were low anterior rectal resections (LARs) with primary anastomosis. In total, 25,824 patients received this type of surgery, with only a small variation between years (highest caseload in 2018: *n* = 5331; lowest caseload of 4914 in 2020). The vast majority of patients received laparoscopic LAR (*n* = 14,668; 57%), followed by open resection (*n* = 9745; 38%) and robotic resection (*n* = 1411; 5%). From 2016 to 2020, the numbers of open LAR steadily declined from 51% in 2016 to 27% in 2020. Inversely, the numbers of laparoscopic and robotic LAR increased from 2016 (47% and 2%, respectively) to 2020 (63% and 10%, respectively). A total of 444 (1.7%) patients undergoing LAR died during hospital stay. Mortality after open LAR was significantly higher in comparison to laparoscopic or robotic LAR (open vs. laparoscopic LAR: 2.5% vs. 1.3%; *p* < 0.0001; open versus robotic: 2.5% vs. 1%; *p* = 0.000442). No significant difference between laparoscopic and robotic LAR was observed (1.3% vs. 1%; *p* = 0.361) (Table 2). 

In-hospital mortality of the whole cohort (*n* = 68,112) was 2.95% (*n* = 2012). A separate analysis of each of the five years revealed patients undergoing open or converted-to-open surgery had a significantly higher probability to die during hospital stay compared to both minimal-invasive approaches (open: 4.61%; laparoscopic: 1.77%; robotic: 1.14% and converted-to-open: 3.95%) (Table 3).

The percentage of in-hospital deaths after open rectal cancer surgery remained stable over the years (lowest in 2016: 4%, and highest in 2018: 5%). The same is true for the two minimal-invasive approaches. Converted-to-open patients had a higher mortality compared to complete minimal-invasive operations, and in all five years, in-hospital deaths were significantly more evident in the converted-to-open group. Compared to planned open surgery patients, patients who were converted-to-open did not die significantly more often during hospital stay. Only in 2018, patients who were converted-to-open surgery did better than patients after planned open surgery (*p* = 0.0423). 

The conversion rate was 2.53% (*n* = 133/5263) in the robotic and 11.94% (*n* = 3874/32,442) in the laparoscopic group, and was highly significant to the favor of the robotic group (*p* < 0.00001).

## 4. Discussion

Minimal-invasive approaches for the treatment of rectal and colon cancer have been intensively investigated in the past decades. Initial concerns emphasized the possibility of tumor cell spread due to manipulation with laparoscopic instruments. A variety of randomized, as well as nonrandomized, trials in colon and rectal cancer patients undergoing minimal-invasive approaches showed comparable oncologic long-term results [4,9,10,11,12,13,14,15,16,17,24,25,26,27,28,29,30,31,32,33]. Furthermore, with surgical and technical improvements in laparoscopic and robotic rectal cancer surgery, sphincter-preserving procedures became possible, especially in the narrow and deep male pelvis, and obese patients. Limiting technical aspects of laparoscopy (decreased depth perception, limited view in critical anatomical areas, assistant-dependent and nonangulable rigid instruments) were overcome by the robotic approach [21]. This is reflected by the fact that with the robotic approach, intersphincteric resections for ultralow rectal cancer can be performed with adequate oncologic safety [34,35]. Apart from the oncologic point of view, the robotic approach for rectal cancer seems to have a variety of other crucial advantages, even over the laparoscopic approach: reduced complication rate [35]; shorter operative time, lower conversion rate and comparable morbidity [30]; decreased conversion rate [28,33,36]; earlier return to bowel function, shorter hospitalization and equivalent morbidity and mortality [33]. On the other hand, the ROLARR trial, published in 2017, did not significantly demonstrate a reduction of conversion-to-open surgery when comparing laparoscopic and robotic surgery [37]. One possible explanation for these results may be the fact that some surgeons, who were experienced in laparoscopic surgery, had not completed their robotic learning curve. 

Unfortunately, the vast majority of publications dealing with this topic do not provide information on in-hospital mortality, comparing the open, the laparoscopic and the robotic approach in patients undergoing rectal cancer surgery. The COLOR-II trial [25] and the ROLARR trial [37] reported mortality within 28 and 30 days, respectively. No information is given on the true in-hospital mortality. In the COLOR-II trial, 1% of patients after laparoscopic, and 2% of patients after open surgery (*p* = 0.409), died within 28 days after operation. In the ROLARR trial, two patients, each in the laparoscopic and robotic surgery group, died within 30 days of surgery. When interpreting this very low number of short-term postoperative deaths, it must be noted that all patients had to meet quite strict study inclusion criteria, and these patients’ cohorts certainly do not reflect the clinical reality. In our nationwide analysis, including the years from 2016 to 2020, 68,112 patients undergoing elective rectal cancer surgery were included. In total, there were 28 different surgical procedures, according to the respective OPS-codes, and patients could be divided into open, laparoscopic, robotic, converted-to-open and sphincter-preserving and non-sphincter-preserving surgery. Regardless of the different groups, the total in-hospital mortality was 2.5% (*n* = 2012). In all analyzed years, the statistical probability to die during hospital stay was the highest for the open surgery group, and the lowest for the robotic surgery group. Even more, there was a statistically significant difference between the laparoscopic and the robotic group (1.77% vs. 1.14%; *p* = 0.001). The robotic approach seems to further minimize the risk of conversion, as depicted above. We subanalyzed the patients who were converted to an open procedure with complete minimal-invasive operated patients. In all included years, patients who were converted to open surgery did significantly worse regarding in-hospital mortality. This emphasizes the advantage of the robotic approach to further minimize the operative trauma and conversion rate, thus avoiding in-hospital death in this patient cohort. The conversion rate in the robotic group was in total 2.53%, and was highest in 2018, with 3% (*n* = 30/995). This is much less than in the ROLARR trial with a conversion in the robotic group of 8.1%, which may be further evidence for an incomplete learning curve [37]. In our analysis, the conversion rate in the laparoscopic group was 11.94% (*n* = 3874/32,442), and was statistically significantly higher than in the robotic group (11.94% vs. 2.53%; *p* < 0.00001). These results are in line with the data provided in the ROLARR trial, with a conversion rate in the laparoscopic arm of 12.2%. The facts that converted-to-open patients had an elevated in-hospital mortality, and that the conversion rate in the robotic group was significantly lower than in the laparoscopic group, make robotic-assisted surgery even more appealing, especially in the setting of anticipated difficult procedural steps in selected patients in laparoscopy. Yamaoka et al. [38] evaluated the robotic approach in patients with locally advanced (cT4a/b) rectal cancers, and showed very satisfying short- and midterm results. No patients required conversion to open surgery, and the incidences of postoperative complications (>Clavien–Dindo III) was only 3.5%. In total, 4.9% of patients had a positive resection margin. These data outline that patients with large and invading rectal cancers should be denied the robotic approach. 

Interestingly, the number of planned robotic non-sphincter-preserving surgeries increased steadily over the years, and in 2020, nearly half of all robotic surgeries (*n* = 969/1739) were planned non-sphincter-preserving. This has to be seen in the spotlight of cost-effectiveness and the operative advantages of the robotic system. Quite obviously, the preparation in the deep pelvis for low rectal cancer can be easier performed with the robotic system. However, the biggest advantage of the robotic approach, namely a safe anastomosis, appears irrelevant in this patient subset. Simianu et al. analyzed the cost-effectiveness of laparoscopic versus robotic minimal-invasive colectomy. The conclusion was that robotic colectomy becomes cost-effective if robotic disposable instruments costs decrease below USD 1341 per case, robotic operating time falls below 172 min or the robotic hernia rate is less than 5% [22]. The increasing availability of robotic systems and additional companies coming into the market will surely lead to a reduction of costs for each procedure. Recently, Diez-Barroso et al. [39] reported a 1.5% robotic hernia rate in the general surgery population, emphasizing the very low postoperative hernia rate after robotic-assisted surgeries. 

## 5. Conclusions

This nationwide analysis showed possible advantages that the robotic resection for rectal cancer might have. Operations with the robotic system seem to be associated with the lowest conversion and in-hospital mortality rate in comparison to open surgery and the laparoscopic approach. A prerequisite is quite obviously a completed learning curve. The limitations of this work are also quite obvious. The data were not harvested from a prospectively maintained clinical database, thus lacking many of treatment- and patient-related parameters. An explanation for the reduced in-hospital mortality for the robotic approach might be that potentially sicker patients were primarily scheduled for the open approach, and that younger and fitter patients were more represented in minimal-invasive surgeries. 

The significantly reduced conversion rate in favor of the robotic approach is another appealing issue, and might extend its applicability to patients with anticipated difficult procedural steps.

Nevertheless, these data reflect the clinical reality in Germany, and should be discussed in order to gain more acceptance for this, so far, expensive surgical approach, because the patients we treat might benefit. 

## Figures and Tables

**Table 1:** Patient characteristics.

Parameter/Year	2016	2017	2018	2019	2020
Total number	13,362	13,520	14,101	14,090	13,039
Sex (male) (%)	8521 (64)	8500 (63)	8875 (63)	9021 (64)	8307 (64)
Operative procedures *n* (%)					
Sphincter-sparing	10,755 (80)	10,692 (79)	11,165 (79)	10,929 (78)	9911 (76)
Non-sphincter-sparing	2607 (20)	2828 (21)	2936 (21)	3161 (22)	3128 (24)
Operative approach *n* (%)					
Open	6847 (51)	5951 (44)	5380 (38)	4608 (33)	3747 (29)
Laparoscopic	5397 (41)	6213 (46)	6832 (49)	7187 (51)	6813 (52)
Robotic	431 (3)	616 (5)	995 (7)	1482 (11)	1739 (13)
Converted-to-open	687 (5)	740 (5)	894 (6)	813 (5)	740 (6)

**Table 2:** Characteristics of patients with deep anterior rectal resection with primary anastomosis (* *p* significant < 0.05).

Parameter/Year	2016	2017	2018	2019	2020	Total
Total number *n* (%)	5248 (39)	5205 (39)	5331 (38)	5126 (36)	4941 (38)	25,824 (38)
Operative approach *n* (%)						
Open	2683 (51)	2234 (43)	1980 (37)	1521 (30)	1327 (27)	9745 (38)
Laparoscopic	2458 (47)	2800 (54)	3072 (58)	3222 (63)	3116 (63)	14,668 (57)
Robotic	107 (2)	171 (3)	279 (5)	383 (7)	471 (10)	1411 (5)
In-hospital mortality *n* (%)						
Open	58 (2.1)	63 (2.8)	44 (2.2)	45 (2.96)	33 (2.49)	243 (2.5)
Laparoscopic	37 (1.5)	35 (1.25)	35 (1.1)	46 (1.43)	34 (1.09)	197 (1.3)
Robotic	0 (0)	0 (0)	3 (1)	6 (1.57)	5 (1)	14 (1)
Statistics for in-hospital mortality						
Open vs. laparoscopic						*p* < 0.00001 *
Open vs. robotic						*p* < 0.00044 *
Laparoscopic vs. robotic						*p* = 0.361

**Table 3:** In-hospital mortality and conversion rates according to year and operative approach (* *p* significant < 0.05).

Parameter/Year	2016	2017	2018	2019	2020	Total
In-hospital mortality *n* (%)						
Open	285 (4)	263 (4)	271 (5)	227 (5)	176 (5)	1222 (5)
Laparoscopic	113 (2)	124 (2)	108 (2)	122 (2)	106 (2)	573 (2)
Robotic	4 (1)	6 (1)	11 (1)	22 (1)	17 (1)	60 (1)
Converted-to-open	32 (5)	36 (5)	31 (4)	31 (4)	23 (3)	154 (4)
Statistics						
Open vs. laparoscopic						*p* < 0.00001 *
Open vs. robotic						*p* < 0.00001 *
Open vs. converted						*p* = 0.065 *
Laparoscopic vs. robotic						*p* = 0.001 *
Conversion rate *n* (%)						
Laparoscopic	687 (13)	740 (12)	894 (13)	813 (11)	740 (11)	3874 (12)
Robotic	14 (3)	17 (3)	30 (3)	26 (2)	46 (3)	133 (3)

## Data Availability

Standort Berlin-Statistisches Bundesamt (destatis.de). Available online: https://www.destatis.de/DE/Ueber-uns/Leitung-Organisation/Standorte/berlin.html (accessed on 1 July 2022).
